# A phase II, multicenter, open-label trial to evaluate the safety and efficacy of ISU303 (Agalsidase beta) in patients with Fabry disease

**DOI:** 10.1097/MD.0000000000030345

**Published:** 2022-09-16

**Authors:** Soojin Hwang, Beom Hee Lee, Woo-Shik Kim, Dae-Seong Kim, Chong Kun Cheon, Chang Hwa Lee, Yunha Choi, Jin-Ho Choi, Ja Hye Kim, Han-Wook Yoo

**Affiliations:** a Department of Pediatrics, Asan Medical Center Children’s Hospital, University of Ulsan College of Medicine, Seoul, Korea; b Medical Genetics Center, Asan Medical Center Children’s Hospital, University of Ulsan College of Medicine, Seoul, Korea; c Department of Internal Medicine, Kyung Hee University College of Medicine, Seoul, Korea; d Department of Neurology, Pusan National University Yangsan Hospital, Pusan National University School of Medicine, Yangsan, Korea; e Department of Pediatrics, Pusan National University Yangsan Hospital, Pusan National University School of Medicine, Yangsan, Korea; f Department of Internal Medicine, Hanyang University Medical Center, Seoul, Korea.

**Keywords:** agalsidase beta, enzyme replacement therapy, Fabry disease

## Abstract

Fabry disease (FD) is caused by a deficiency in the activity of the lysosomal enzyme, α-galactosidase A (α-Gal A), which leads to globotriaosylceramide (Gb3) deposition in multiple tissues. The current management of FD is enzyme replacement therapy (ERT). We report on the efficacy and safety of a new agalsidase beta, ISU303, in FD.

**Methods:**

Ten patients (7 males, 3 females) were enrolled and administered a 1 mg/kg dose of ISU303, every other week for 6 months. The primary endpoint was the normalization of plasma Gb3 level. The secondary endpoints were the changes from baseline in urine Gb3 and the plasma and urine lyso-globotriaosylsphingosine (lyso-Gb3) level. Echocardiography, renal function test, and pain-related quality of life were also assessed before and after administration. Safety evaluation was performed including vital signs, laboratory tests, electrocardiograms, antibody screening tests, and adverse events at each visit.

**Results:**

At 22 weeks of treatment, plasma and urine Gb3 level decreased by a mean of 4.01 ± 1.29 μg/mL (range 2.50–5.70) (*P *= .005) and 1.12 ± 1.98 μg/mg Cr. (range 0.04–5.65) (*P *= .017), respectively. However, no significant difference was observed in plasma and urine lyso-Gb3 levels. Echocardiography also was not changed. Renal function and pain-related quality of life showed improvements, but there was no clinical significance. No severe adverse events were observed. Only 1 patient developed an anti-drug antibody without neutralizing activity during the trial.

**Conclusion:**

This study showed the efficacy and safety of ISU303. Treatment with ISU303 significantly resulted in plasma and urine Gb3 decrease in patients with FD. These results suggest that ISU303 is safe and effective and can alternative ERT for FD.

## 1. Introduction

Fabry disease (FD, OMIM #301500), a rare X-linked lysosomal storage disorder, is caused by mutations in the *GLA* gene encoding α-galactosidase A (α-Gal A). The functional deficiency of α-Gal A accumulates glycosphingolipids, including globotriaosylceramide (Gb3) and globotriaosylsphingosine (lyso-Gb3) in cells, which lead to tissue damage and progress to multi-organ failure involving the kidney, heart, and nervous systems.^[1]^

The prevalence of FD, an X-linked disorder, is estimated at 1 in 40,000 to 1 in 117,000 males.^[2,3]^ Classic FD, who are mostly males with the absence of α-Gal A activity, are developed in early childhood and have severe clinical symptoms. Patients often experience multisystem symptoms such as acroparesthesia, hypohidrosis, angiokeratoma, corneal opacity, hearing loss, and progress to renal failure, cardiovascular disease, and stroke.^[4,5]^ FD also occurs in females with classic or nonclassical types.^[6]^ Non-classical FD are late-onset and mild phenotypes, who are with decreased but some residual α-Gal A activity.^[1,7,8]^

Enzyme replacement therapy (ERT) with recombinant human α-Gal A significantly reduces Gb3/Lyso-Gb3 accumulation and improves the clinical outcomes of FD.^[9,10]^ There are 2 preparations approved: algalsidase alfa (Replagal^®^; Shire Human Genetic Therapies AB, Danderyd, Sweden) and agalsidase beta (Fabrazyme^®^; Genzyme Corporation, Cambridge, MA). The efficacy and safety of ERT have been validated by numerous studies.^[11]^ In addition, the pharmacologic chaperone Migalastat (Galafold^®^; Amicus Therapeutics, Cranbury, NJ) has been also developed for FD patients with amenable mutations.^[12]^ However, those agents still infer some considerations regarding optimal treatment timing, dose, therapeutic goals, and high costs.

The other form of agalsidase beta, ISU303, is newly developed by ISU Abxis (Seongnam, Korea). ISU303 is made by recombinant DNA technology in the Chinese Hamster Ovary (CHO) cell line, in the same manner as Fabrazyme^®^.^[13]^ Both enzymes have almost identical structures which are glycoproteins containing sialic acid and mannose-6-phosphate residues. They bind mannose-6-phosphate receptor and activate the same signaling pathway, resulting in similar physicochemical, biological, and pharmaceutical features.^[14]^ The phase I clinical trial was completed in 18 healthy subjects, which demonstrated tolerability and safety of ISU303.^[15]^ In this study, we report on phase II clinical trial to evaluate the efficacy and safety of ISU303 in 10 Korean patients with FD.

## 2. Methods

### 2.1. Study design

This multicenter, open-label, phase II study was conducted to evaluate the efficacy, safety, and pharmacokinetics (PK) of ISU303 in patients with FD. Eligible FD patients were as follows: diagnosis of FD by α-Gal A activity assay or GLA gene mutation; age > 16 years; the presence of more than 1 symptom caused by FD. The exclusion criteria were as follows: renal replacement therapy or kidney transplantation; hypersensitivity to agalsidase beta; critical illness not related to FD; pregnancy or breastfeeding; treatment with an investigational drug within 30 days before enrollment. The study drug ISU303 is a recombinant human α-galactosidase produced from CHO cells line using recombinant DNA technology. The structural, physicochemical, and biological characteristics of ISU303 were similar to agalsidase beta (Fabrazyme^®^) approved by the US Food and Drug Administration in 2003. In the phase I clinical trial, 1 mg/kg of ISU303 was increased gradually, proving its safety and tolerance. Written informed consent was obtained from all patients. Eligible patients, regardless of fasting state, received 1 mg/kg of ISU303 by intravenous infusion every 2 weeks for 6 months. ISU303 was reconstituted in 500 mL of normal saline and then infused at a rate of 15 mg/h over 4 to 6 hours. This study was approved by the Institutional Review Boards (IRB) of each hospital (Asan Medical Center, IRB No. 2012-0079; Kyung Hee University College of Medicine, IRB No. KMC IRB 1203-04; Pusan National University Yangsan Hospital, IRB No. 02-2012-004; Hanyang University Medical Center, IRB No. HYUH 2012-02-013) and conducted according to the Good Clinical Practice and in agreement with the principles of the Helsinki declaration.

### 2.2. Efficacy assessments

Fourteen study visits were scheduled: screening (visit 0), administration (biweekly visits 1–12; week 0–22), and study termination visit (week 24). The primary efficacy was evaluated as to whether plasma Gb3 reached and maintained a normal range or less at week 22. Secondary efficacy variables included changes in other biomarkers (urine Gb3, lyso-Gb3) and clinical outcomes (cardiac and renal function, pain, and quality of life) from baseline to week 22. Gb3 and lyso-Gb3 measurement was performed using a validated liquid chromatography-tandem mass spectrometry. Echocardiography and 24-hour urine collection were done. The glomerular filtration rate was estimated by CKD-EPI (Chronic Kidney Disease Epidemiology Collaboration) equation. Pain intensity measured by Short-Form McGill Pain Questionnaire. Quality of life was assessed by SF-36 Health Status Survey and subject symptoms diary.

### 2.3. Safety assessments

Safety evaluations were performed at each visit, including adverse events, vital signs, physical examination, and laboratory tests (hematology, serum chemistry, and urinalysis). Anti-drug antibody and neutralizing activity were measured at week 0, 4, 10, 16, and 22. For screening anti-drug antibody (immunoglobulin G) in serum from FD patients, enzyme-linked immunosorbent assay plates coated with agalsidase beta (Fabrazyme, Sanofi Genzyme, Cambridge, MA) were used, as described previously.^[15]^ To examine neutralizing activity, the serum-mediated α-Gal inhibition test was performed. This assay is based on the hydrolysis of the substrate 4-methylumbelliferyl-α-D-galactopyranoside (Sigma, St. Louis, MO) to 4-methyl-umbelliferone, as previously described with slight modifications.^[16]^

### 2.4. Pharmacokinetics

For PK analysis, blood samples were collected before administration (0 hours), at 1, 3, 5 hours during infusion, and at 6, 6.5, 7, 8, 10 hours after the initiation of infusion. The blood concentration-time curve analysis was performed by linear and log-linear models. The PK of ISU303 were determined by noncompartmental analysis using the Phoenix WinNonlin version 6.3 (Pharsight, CA, USA) program. The following PK parameters were calculated: area under the curve from the time of dosing to the last measurable concentration (AUC_last_), maximal concentration of drug (*C*_max_), time to reach *C*_max_, half-life, and serum clearance. In addition, *C*_max_ and AUC_last_ were adjusted for the dosage compared to the PK results of phase I clinical study.^[15]^

### 2.5. Statistical analysis

Descriptive statistics are presented as means ± standard deviations. Wilcoxon signed-rank test was used to evaluate the parameters. All tests were 2-sided, and a *P* value of <.05 (95% confidence) was considered statistically significant. Analyses were performed with SPSS version 27.0.

## 3. Results

**Figure 1.**
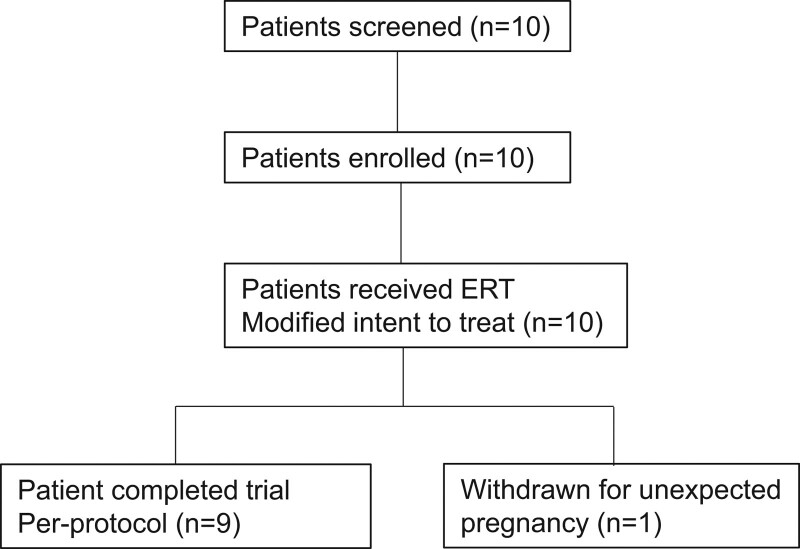
Study flow.

Ten Korean patients with FD were enrolled at 4 study centers in South Korea. All patients were assessed with the primary endpoints at least once and included in the efficacy and safety analysis group. However, only 9 patients completed the study because 1 patient was withdrawn due to unexpected pregnancy. Efficacy analysis was conducted for the modified intention-to-treat (MITT) analysis group and per-protocol (PP) group (Fig. 1). The results were described below to use MITT analysis group data.

### 3.1. Baseline characteristics of patients

Table 1 provides demographic and baseline characteristics. Among the 10 patients with FD, 7 were male and 3 were female. The mean age at treatment initiation was 33 ± 12 years (range 21–56) and the mean age at diagnosis was 29 ± 13 years (range 16–53). Eight patients had received previous ERT with Fabrazyme^®^ for 4.6 ± 3.2 years (range 1–9). The other 2 patients were newly diagnosed and started treatment with ISU303. All patients had GLA gene mutations. c.1235_1236del (p.Thr412Serfs*38) and c.861G>A (p.Trp287*) were recurrently found in 2 patients, respectively. Nine patients were confirmed by the decrease of α-Gal A activity in leukocytes or plasma, whereas it was not measured in the remaining 1 female patient (patient No. 10). Classic phenotypes in 8 patients (7 males, 1 female) were early-onset and affected multiorgan systems (patients No. 1–4 and 6–9). Patients generally experienced neuropathic pain and autonomic nervous system dysfunction such as acroparesthesia and hypohidrosis from early childhood and progressed to multiorgan damage including heart (left ventricular hypertrophy and arrhythmias) and kidney (proteinuria and decline in glomerular filtration rate). Non-classical phenotypes in 2 females (patients No. 5 and 10) were late-onset and less severe clinical manifestations. Overall, the most common symptoms were acroparesthesia (9/10 patients) and hypohidrosis (8/10 patients) related to nerve systems. Left ventricular hypertrophy was observed in 4 patients and overt proteinuria was also found in 5 patients. At start of treatment, mean plasma Gb3 was 8.1 ± 1.6 μg/mL (normal, <9.9) and urine Gb3 was 1.4 ± 2.4 μg/mg Cr. (normal, <0.9). The mean of lyso-Gb3 was 20.02 ± 14.29 ng/mL in plasma (normal, <1.74), while lyso-Gb3 in urine was not detected (normal, not detected) in all patients.

**Table 1:** Patient demographics and baseline characteristics (n = 10).

No.	Sex	Age (yr)	Height (cm)	Weight (kg)	Age at diagnosis (yr)	Genotype	α-Gal a activity (nmol/h/mg)	Clinical symptoms	Previous ERT	Duration of ERT (yr)	Plasma Gb3 (μg/mL)	Urine Gb3 (μg/mg Cr.)	LVMI (g/m^2^)	Proteinuria (mg/d)	eGFR (mL/min/1.73 m^2^)
1	M	49	171.1	67.5	46	c.966C>G	Leukocyte	LVH, right bundle branch block, acroparesthesia, hypohidrosis, heat intolerance, psoriasis, corneal opacity, tinnitus, hearing loss, vertigo	Fabrazyme	3	11	0.09	275.1	90	94
						(p.Asp322Glu)	0.72								
2	M	28	165.9	78.4	19	c.1235_1236del	Plasma	LVH, bradycardia, 1st degree AV block, proteinuria, acroparesthesia, hypohidrosis, heat intolerance, angiokeratoma, cataract, tinnitus	Fabrazyme	9	7.2	0.33	127.3	2029.7	90
						(p.Thr412Serfs*38)	0								
3	M	23	176.6	61.6	16	c.1235_1236del	Leukocyte	Bradycardia, WPW syndrome, acroparesthesia, hypohidrosis, heat intolerance, cornea verticillata, myopia with astigmatism, tinnitus	Fabrazyme	7	7.5	0.13	92.11	60	121
						(p.Thr412Serfs*38)	0								
4	M	21	174.4	63	16	c.426C>G	Leukocyte	Bradycardia, acroparesthesia, hypohidrosis, angiokeratoma, myopia	Fabrazyme	5	7.5	0.1	80.2	110.1	134
						(p.Cys142Trp)	0								
5	F	44	154.7	74.2	44	c.928C>G	Leukocyte	Proteinuria, acroparesthesia, hypohidrosis, angiokeratoma, hearing loss	-	0	7.8	0.05	81.5	32.6	94
						(p.Leu310Val)	31.6								
6	M	26	168	60	24	c.861G>A	Leukocyte	Acroparesthesia, hypohidrosis	Fabrazyme	1	10.8	7.37	76.5	52	109
						(p.Trp287*)	3								
7	M	23	167	56	21	c.861G>A	Leukocyte	Proteinuria, acroparesthesia, hypohidrosis	Fabrazyme	1	8.5	4.09	645.5	235	159
						(p.Trp287*)	2.4								
8	M	32	172	74.9	24	c.982G>A	Plasma	LVH, proteinuria	Fabrazyme	7	7.1	0.54	104.4	1123.2	74
						(p.Gly328Arg)	0.01								
9	F	28	161.2	49.4	27	c.676T>G	Plasma	Proteinuria, acroparesthesia, hypohidrosis	-	0	6	1.08	52.7	286	127
						(p.Trp226Gly)	0.12								
10	F	56	165.8	71.9	53	c.611-11T>A	NA	LVH, atrial fibrillation, proteinuria, acroparesthesia, corneal opacity	Fabrazyme	1	7.6	0.06	121.4	178.1	68

Normal range of α-gal A activity in leukocyte > 37 nmol/h/mg; in plasma > 4 mmol/h/mg. LVH (left ventricular hypertrophy) was defined as LVMI (left ventricular mass index) > 95 g/m^2^ for females and >115 g/m^2^ for males. Proteinuria was defined as >150 mg/d.

eGFR = estimated glomerular filtration rate, ERT = enzyme replacement therapy, Gb3 = globotriaosylceramide, NA = not available, WPW = Wolff-Parkinson-White syndrome.

### 3.2. Efficacy of ISU303

#### 3.2..1. Plasma Gb3 level.

**Figure 2.**
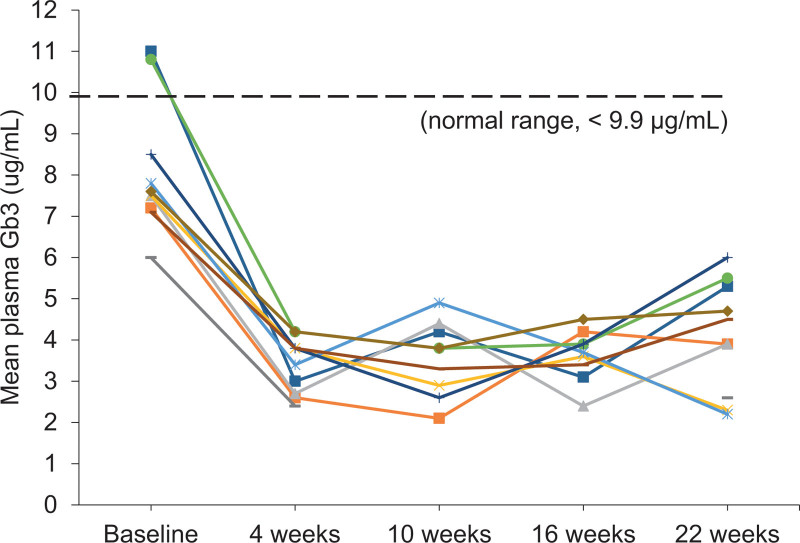
Plasma Gb3 over time. Individual patient data and normal range (n = 10). Gb3 = globotriaosylceramide.

The primary endpoint in our study was to reach a normal range of plasma Gb3 at 22 weeks of treatment with ISU303. The mean plasma Gb3 at baseline was 8.1 ± 1.6 μg/mL (range 6.00–11.0); it decreased significantly to 4.09 ± 1.36 μg/mL (range 2.20–6.00) after 22 weeks of treatment (*P *= .005). The 95% confidence interval for the mean of plasma Gb3 was 3.21 and 5.06 μg/mL (Table 2). This result was at a normal range below 9.9 μg/mL, which demonstrated the efficacy of ISU303 (Fig. 2). In addition, the mean of plasma Gb3 was 8.33 ± 1.51 μg/mL (range 7.10–11.0) at baseline and decreased to 4.26 ± 1.33 μg/mL (range 2.20–6.00) at week 22 in the PP analysis group (*P *= .008).

**Table 2:** Plasma Gb3 level (μg/mL).

Visit	n	Mean ± SD	Change*	*P* value
Baseline	10	8.10 ± 1.60		
4 wk	10	3.39 ± 0.67	−4.71 ± 1.51	.005
10 wk	9	3.56 ± 0.91	−4.78 ± 1.53	.008
16 wk	9	3.63 ± 0.62	−4.70 ± 1.68	.008
22 wk	10	4.09 ± 1.36	−4.01 ± 1.29	.005

Gb3 = globotriaosylceramide.

* The value change at 22 wk compared to the baseline.

#### 3.2..2. Urine Gb3, plasma, and urine lyso-Gb3 level.

On treatment, mean urine Gb3 level declined from 1.38 ± 2.44 μg/mg Cr. (range 0.05–7.37) to 0.27 ± 0.52 μg/mg Cr. (range 0.02–1.72), which satisfied the normal range below 0.90 μg/mg Cr. (*P *= .11). In the case of lyso-Gb3, mean plasma lyso-Gb3 decreased from 20.02 ± 14.29 μg/mL (range 0.00–46.66) at baseline to 18.03 ± 16.13 μg/mL (range 0.00–54.32) at week 22 (*P *= .13). There was also no significant change in urine lysoGb3. The mean was 0.00 ± 0.00 μg/mg Cr. at baseline and 0.88 ± 2.78 μg/mg Cr. (range 0.00–8.80) at week 22 (*P *= 1.00) (Table 3).

**Table 3:** Urine Gb3, plasma, and urine lyso-Gb3 level.

	n	Mean ± SD		Change*	*P* value
		Baseline	22 wk		
Urine Gb3 (μg/mg Cr.)(normal range, <0.9)	10	1.38 ± 2.44	0.27 ± 0.52	−1.12 ± 1.98	.017
Plasma lyso-Gb3 (ng/mL)(normal range, <1.74)	10	20.02 ± 14.29	18.03 ± 16.13	−1.99 ± 4.05	.128
Urine lyso-Gb3 (μg/mg Cr.)(normal range, not detected)	10	0.00 ± 0.00	0.88 ± 2.78	0.88 ± 2.78	1.00

Gb3 = globotriaosylceramide.

* The value change at 22 wk compared to the baseline.

#### 3.2..3. Cardiac function.

Cardiac function was assessed by echocardiogram. Echocardiographic values are presented in Table 4. From baseline to week 22, there was no significant change in left ventricular mass index (LVMI) and left ventricular posterior wall thickness. The mean of LVMI was 107.66 ± 63.31 g/m^2^ (range 52.68–275.10) and then was stationary at 111.66 ± 61.74 g/m^2^ (range 48.44–265.40) after 22 weeks of treatment (*P *= .63). left ventricular posterior wall thickness was also not altered by treatment. The mean of LVWd was 11.85 ± 6.00 mm (range 8.00–7.00) at baseline, which was not significantly changed into 11.77 ± 5.27 mm (range 8.00–26.00) at week 22 (*P *= .63).

**Table 4:** Echocardiographic measurements.

	n	Mean ± SD		Change*	*P* value
		Baseline	22 wk		
LVID (mm)	10	46.98 ± 8.70	48.99 ± 4.61	2.02 ± 9.10	.945
LVPWd (mm)	10	11.85 ± 6.00	11.77 ± 5.27	−0.99 ± 2.18	.625
IVSd (mm)	10	9.93 ± 4.68	9.66 ± 4.70	−0.27 ± 1.65	.906
LVMI (g/m^2^)	10	107.66 ± 63.31	111.66 ± 61.74	4.00 ± 13.44	.625
LVEF (%)	10	67.22 ± 6.06	68.20 ± 6.00	0.98 ± 6.40	.555

IVSd = interventricular septum thickness at diastole, LVEF = left ventricular ejection fraction, LVID = left ventricle inner dimension, LVMI = left ventricular mass index, LVPWd = left ventricular posterior wall thickness at diastole.

* The value change at 22 wk compared to the baseline.

#### 3.2..4. Renal function.

Twenty-four hour-collected urine protein excretion and estimated glomerular filtration rate (eGFR) were measured (Table 5). Proteinuria was observed in 5 patients at baseline, which was not significantly different by treatment. The mean was 419.67 ± 651.18 mg/d (range 32.60–2029.70) at baseline and 576.81 ± 894.98 mg/d (range 32.60–2029.70) at week 22 (*P *= .49). The mean of eGFR tended to increase from 107.00 ± 28.42 mL/min/1.73 m^2^ (range 68.00–159.00) to 116.33 ± 29.77 mL/min/1.73 m^2^ (range 77.00–163.00) without significance (*P *= .34).

**Table 5:** Renal function tests.

	n	Mean ± SD		Change*	*P* value
		Baseline	22 wk		
Proteinuria (mg/d)	10	419.67 ± 651.18	576.81 ± 984.98	157.14 ± 379.45	.492
eGFR (mL/min/1.73 m^2^)	10	107.00 ± 28.42	116.33 ± 29.77	5.00 ± 16.78	.344

eGFR = estimated glomerular filtration rate.

* The value change at 22 wk compared to the baseline.

#### 3.2..5. Pain severity and quality of life.

Pain and pain-related quality of life in FD are summarized in Table 6. The results of the Short-Form McGill Pain Questionnaire, subject symptom diary, and SF-36 Health Status Survey showed no significant difference after treatment. Regarding pain severity, the mean total score in the Short-Form McGill Pain Questionnaire decreased from 13.10 ± 11.48 (range 0.00–40.00) at baseline to 9.80 ± 7.58 (range 0.00–28.00) at week 22 (*P *= .19). Subject symptoms diary scores also decreased from 7.70 ± 4.62 (range 0.00–15.00) to 7.30 ± 4.11 (range 0.00–13.00) (*P *= .63). These results suggested that the treatment tends to reduce pain in patients. However, the quality of life for the patients using the SF-36 Health Status Survey was stationary. The mean total score was 60.59 ± 16.57 (range 35.43–88.57) at baseline and 60.93 ± 18.42 (range 31.86–88.57) at week 22 (*P *= .84).

**Table 6:** Pain and quality of life.

	n	Mean ± SD		Change*	*P* value
		Baseline	22 wk		
Short Form McGill Pain Questionnaire	10	13.10 ± 11.48	9.80 ± 7.58	−3.30 ± 6.62	.186
Subject symptom diary	10	7.70 ± 4.62	7.30 ± 4.11	−0.40 ± 2.41	.625
SF-36 Health Status Survey	10	60.59 ± 16.57	60.93 ± 18.42	0.34 ± 9.94	.840

* The value change at 22 wk compared to the baseline.

### 3.3. Safety

No serious event was reported in this study. There was no adverse event to lead to discontinuation of treatment. Nine patients experienced adverse events with 43 cases in total (Table 7). Among the reported adverse events, the drug-related adverse events were 10 cases in 4 patients (40%). General disorders including pyrexia and chills, and nervous system disorders were common. All events were mild to moderate.

**Table 7:** Adverse drug reactions.

	ISU303 (n = 10)		
	n [cases]		
		Mild	Moderate
Total no. of adverse events	9 [43]		
Total no. of adverse drug reactions	4 [10]		
Nervous system disorders	2 [3]	-	-
Dizziness	1 [1]	-	1 [1]
Dysgeusia	1 [1]	1 [1]	-
Somnolence	1 [1]	-	1 [1]
Gastrointestinal disorders	1 [1]	-	-
Vomiting	1 [1]	1 [1]	-
General disorders	2 [5]	-	-
Pyrexia	1 [4]	1 [4]	-
Chills	1 [1]	1 [1]	-
Skin disorders	1 [1]	-	-
Pruritis	1 [1]	1 [1]	-

Five of the 10 patients already had anti-drug antibodies (ADA) at baseline, 3 of which had neutralizing activity. During the treatment, the development of ADA without neutralizing activity was observed in 1 patient at week 10 (Table 8). The ADA without neutralizing activity appeared to have no significant effect on the safety and efficacy of ISU303.

**Table 8:** Anti-drug antibodies.

	ISU303 (n = 10)				
	Baseline	4 wk	10 wk	16 wk	22 wk
Antibody formation (neutralizing antibody)	5 (3)	5 (3)	6 (3)	6 (3)	6 (3)

### 3.4. Pharmacokinetics of ISU303

PK analysis of ISU303 is presented in Table 9. After a single intravenous infusion of ISU303 1 mg/kg with 15 mg/h, the mean of *C*_max_ was 290.5 ± 128.1 mU/mL (range 171.5–601.3) and the median of time to *C*_max_ was 5 hours (range 1–8). The mean of AUC_last_, serum clearance, and half-life were 1048.6 ± 569.1 h·mU/mL, 77.5 ± 17.9 mL/h/kg, and 1.13 ± 0.34 h, respectively. However, 2 out of 3 patients who already had ADA with neutralizing activity at baseline showed quite different patterns of PK. One patient (patient No. 6) did not increase drug concentration during the infusion but finally increased it 8 hours later. The other patient (patient No. 7) showed 2 peak concentration times at 8 and 10 hours after infusion.

**Table 9:** Pharmacokinetic parameters.

	ISU303 1 mg/kg				
	*C*_max_ (mU/mL)	*T*_max_ (h)	AUC_last_ (h·mU/mL)	CL (mL/h/kg)	*t*_1/2_ (h)
n	10	10	10	8*	8*
mean	290.5	4.6	1048.6	77.5	1.13
SD	128.1	1.9	569.1	17.9	0.34
min	171.5	1.0	472.5	51.9	0.92
median	238.7	5.0	941.5	74.1	1.02
max	601.3	8.0	2530.5	104.1	1.96

AUC_last_ = area under the curve from the time of dosing to the last measurable concentration, *C*_max_ = maximal concentration of drug, CL = serum clearance, FD = Fabry disease, *T*_max_ = time to reach *C*_max_, *t*_1/2_ = half-life.

* PK parameters were not calculated in 2 patients with neutralizing anti-drug antibodies (patient No. 6 and 7).

## 4. Discussion

This study showed the results of phase II, multicenter, open-label trial to evaluate the efficacy and safety of ISU303 in patients with FD. Treatment with ISU303 significantly reduced plasma Gb3 and had tolerable adverse events during the observation period. In addition, it stabilized the disease progression regarding renal and cardiac dysfunction and pain severity.

The primary efficacy analysis was assessed whether ISU303 reduced plasma Gb3 during the treatment. There was a significant decrease in plasma Gb3 in both MITT and PP analysis groups (*P* < .01). All patients showed to reach a normal range of plasma Gb3 at week 4 and were stabilized at week 22. This result demonstrated the efficacy of ISU303 and at least similar effectiveness to previous ERT.

Because of the small number of patients with rare diseases, the study population included 8 patients who were switched from previous ERT (Fabrazyme^®^) and 2 treatment naïve patients. Regardless of previous treatment status, the overall efficacy of ISU303 was proved to decrease plasma Gb3 in patients with FD, as described above. There was no significant difference in plasma Gb3 level between the switchover subgroup and treatment naïve subgroup during the treatment. For switchover group, mean baseline plasma Gb3 level was 8.40 ± 1.60 μg/mL (range 7.10–11.00) and significantly decreased to 4.51 ± 1.16 μg/mL (range 2.30–6.00) at week 22 (*P *= .012). The mean of plasma Gb3 in treatment naïve group was 6.90 ± 1.27 μg/mL (range 6.00–7.80) at baseline and 2.40 ± 0.28 μg/mL (range 2.20–2.60) at week 22 (*P *= .180).

Regarding other efficacy parameters, mean urine Gb3 was decreased to a normal range at 22 weeks of treatment (*P* = .17). Lyso-Gb3 also tended to decrease in plasma level (*P *= .13). Urine lyso-Gb3 was not detected in all patients at baseline. However, only 1 female patient who dropped out due to pregnancy showed 0.88 ± 2.78 μg/mg Cr of urine lyso-Gb3 at week 22. This might be explained by the fact that physiologic stress like pregnancy aggravates disease severity in FD transiently. Recent studies have reported that lyso-Gb3 is strongly associated with disease severity and could be a useful diagnostic marker in FD. Plasma and urine lyso-Gb3 were significantly elevated in symptomatic FD patients.^[17,18]^ ERT also significantly decreased plasma lyso-Gb3 but it is still under debate about urine lyso-Gb3.^[19–21]^

ERT improves clinical symptoms following reduction of Gb3 in patients with FD.^[22]^ The clinical response to ERT may differ depending on disease severity and organ involvement at the start of treatment. In the present study, cardiac and renal function was stable for the treatment with ISU303. Four of 10 patients already had mild to severe left ventricular hypertrophy assessed by LVMI at baseline and no significant change was observed at week 22 (*P *= .63) as in previous studies.^[23,24]^ Five patients showed overt proteinuria, which was with normal to mild kidney dysfunction assessed by eGFR at baseline. At week 22, proteinuria and eGFR did not change significantly. There was no patient in whom renal dysfunction progressed. Effects of agalasidase beta on proteinuria and eGFR were variable depending on baseline proteinuria and chronic kidney disease stages, which might have not changed or reduced the slope of eGFR decline.^[24,25]^ In addition, pain-related quality of life in the patients tended to improve over the study. These results were consistent with previous studies.^[26–28]^

Previous phase I clinical trial in healthy volunteers has demonstrated the safety and PK of ISU303, which was well tolerated at doses up to 1 mg/kg.^[15]^ In this study, all patients received a biweekly intravenous infusion of 1 mg/kg ISU303 for 6 months. This treatment was tolerated without serious adverse events, consistent with phase I clinical data. The most common drug-adverse reaction was pyrexia from 1 patient with 4 cases. The others were somnolence, dizziness, dysgeusia, vomiting, chills, and pruritis in 1 patient with 1 case. Most cases were mild and treatable. Furthermore, only 1 patient newly developed ADA without neutralizing activity after 10 weeks of treatment.

With regard to PK analysis, the plasma concentration of ISU303 continuously increased during the infusion period. The PK profile of ISU303 was similar to healthy subjects with few differences. The mean of *C*_max_ and AUC_last_ were higher than normal healthy subjects (290.5 mU/mL vs 195.9 mU/mL, 1048.6 h·mU/mL vs 940.0 h·mU/mL, respectively). The half-life and clearance in this study were 1.13 hours and 77.5 mL/h/kg, which were slightly decreased compared to those previously reported in healthy people (1.46 hours and 80.4 mL/h/kg, respectively). This discrepancy was probably related to renal function status in FD patients. However, these differences were not clinically significant.

Interestingly, neutralizing ADA might affect the PK of ISU303. Two patients who had previous ERT with Fabrazyme^®^ and neutralizing antibodies showed a slow increase in plasma concentration and delayed time of the maximum concentration of ISU303. These distinctions might be caused by interaction with ADA or individual differences in organ and intracellular metabolism. Furthermore, neutralizing ADA had inhibitory function via directly binding agalasidase beta or modulating its signaling pathway, which probably led to adverse impacts on the PK and efficacy.^[29]^ Previous studies demonstrated that the types and doses of ERT (e.g., agalsidase alfa and agalsidase beta) had different risks and effects on developing ADA in FD.^[30]^ The clinical significance of ADA in FD is still under research. In this clinical trial, the primary efficacy of ISU303 was not significantly altered by ADA.

There were several limitations in this study. The number of patients was small, and the gender ratio was not equal. Most patients had already received previous ERT with Fabrazyme^®^ but only 2 female patients were treatment naïve. The study period was relatively short. More patients and long-term observations are needed in future studies.

ERT used to treat rare, inherited metabolic diseases including Fabry, Gaucher, and Pompe disease has been very expensive until now.^[31]^ A highly similar and comparable to original biologic medicine, has the potential to reduce the cost and improve patient access. This has concomitantly saved the long-term health care cost and facilitated the development of new drugs.^[32,33]^ Development of ISU303 can provide treatment opportunities at a lower cost for patients with FD in many countries. More patients with FD can choose treatment with ISU303 clinically equal to agalsidase beta (Fabrazyme^®^), which may improve patient outcomes. However, it should be considered that these alternative agents are often accompanied by the danger of patent infringement and poor quality. Once biosimilars are introduced, they must require close monitoring to ensure their quality, safety, and efficacy.^[34]^

## 5. Conclusion

The efficacy of this study shows that ISU303 achieved the normal range of plasma Gb3 and the stabilization of clinical outcomes including cardiac, renal function, and quality of life. In addition, ISU303 was well-tolerated in all enrolled patients. Therefore, we anticipate that ISU303 will be comparable to original agalsidase beta and a treatment option for patients with FD.

## Acknowledgments

We appreciate the patients for participating in this study.

## Author contributions

**Conceptualization:** Beom Hee Lee, Han-Wook Yoo.

Formal analysis: Beom Hee Lee.

Investigation: Soojin Hwang, Beom Hee Lee, Woo-Shik Kim, Dae-Seong Kim, Chong Kun Cheon, Chang Hwa Lee, Yunha Choi, Jin-Ho Choi, Ja Hye Kim, Han-Wook Yoo.

Project administration: Han-Wook Yoo.

Supervision: Beom Hee Lee, Han-Wook Yoo.

Writing – original draft: Soojin Hwang.

Writing – review & editing: Soojin Hwang.
